# Achievement of Target Glycemic Goal with Simple Basal Insulin Regimen in Women with Gestational Diabetes: A Prospective Cohort Study

**DOI:** 10.1155/2023/9574563

**Published:** 2023-05-29

**Authors:** Misao Fukuoka, Ichiro Yasuhi, Hiroshi Yamashita, Kensuke Ashimoto, Nao Kurata, Junko Yamaguchi, Megumi Koga, So Sugimi, Sachie Suga, Masashi Fukuda

**Affiliations:** ^1^Department of Obstetrics and Gynecology, National Hospital Organization (NHO) Nagasaki Medical Center, Omura-City, Nagasaki, Japan; ^2^Department of Obstetrics and Gynecology, Kameda General Hospital, Kamogawa, Chiba, Japan

## Abstract

There is little evidence concerning the need to treat gestational diabetes (GDM) in the same way as pregestational diabetes. We evaluated the efficacy of the simple insulin injection (SII) regimen for achieving the target glucose goal without increasing adverse perinatal outcomes in singleton pregnant women with GDM. All subjects underwent self-monitoring of blood glucose (SMBG), and insulin therapy was indicated according to the SMBG profile. Insulin was initially started with the SII regimen, in which one daily injection of NPH insulin before breakfast was used, and another NPH injection was added at bedtime, if necessary. We used the target glucose as <95 mg/dL at fasting and <120 mg/dL postprandial and accepted <130 mg/dL for the latter. If the target glucose did not reach with the regimen, we switched to the multiple daily injection (MDI) with additional prandial insulin aspart. We compared the SMBG profile before delivery as well as the perinatal outcomes between the SII and MDI groups. Among 361 women (age 33.7 years, nullipara 41%, prepregnancy body mass index 23.2 kg/m^2^) with GDM, 59%, 18%, and 23% were in the diet-alone, SII, and MDI groups, respectively. Consequently, regarding women requiring insulin therapy, 43% were treated with the SII regimen throughout pregnancy. The severity of baseline hyperglycemia according to the SMBG data at baseline was the MDI>the SII>the diet group. The rate of achieving target glucose levels before delivery in the SII group at fasting, postprandial < 120 mg/dL and <130 mg/dL were 93%, 54% and 87%, respectively, which were similar to that in the MDI group (93%, 57%, and 93%, respectively), with no significant differences in perinatal outcomes. In conclusion, more than 40% of women with GDM requiring insulin therapy achieved the target glucose goal with this simple insulin regimen without any increase in adverse effects.

## 1. Introduction

Even though gestational diabetes (GDM) is considered a milder form of hyperglycemia than pregestational diabetes [1], various perinatal complications, including macrosomia, primary cesarean section, neonatal hypoglycemia, and preeclampsia, develop without intervention [2–4]. To prevent such perinatal complications, strict glycemic control equivalent to that in normal pregnant women is required, and insulin therapy is indicated if diet-based treatment fails to maintain normoglycemia.

Various types of insulin, including human insulin and insulin analog, can be safely administered to pregnant women. As with pregestational diabetic women, most physicians prefer to administer intensive insulin therapy, such as the multiple daily insulin injection (MDI) regimen, in which basal and rapid acting insulin injections are combined, to women with GDM. However, women with GDM develop less severe hyperglycemia than pregnant women with pregestational diabetes [1]. Furthermore, there is little evidence concerning the need to treat GDM in the same way as pregestational diabetes. In addition, from the patient's perspective, the MDI regimen can cause both physical and mental stress and reduce the quality of life [5]. Recent studies have shown that 20%-60% of women with GDM require insulin therapy to achieve the target glucose goal [3, 6–8]. Thus, for women with GDM, a simpler insulin regimen may help improve compliance of insulin therapy during pregnancy.

We hypothesized that some women with GDM who did not reach target glucose levels with nutrition therapy alone and required insulin therapy had less severe hyperglycemia than those requiring the MDI regimen and could therefore be treated with a simpler regimen than the regimen. For this reason, we developed the simple insulin injection (SII) regimen, in which we use single or twice daily injection of NPH insulin as a treatment option for women with GDM.

The present study evaluated the efficacy of the SII regimen and demonstrated whether it successfully achieved the target glucose goal comparable to the MDI regimen without increasing the risk of adverse perinatal outcomes in women with GDM.

## 2. Materials and Methods

At a single tertiary perinatal care center (National Hospital Organization (NHO) Nagasaki Medical Center, Omura, Nagasaki) in Japan, we prospectively included Japanese singleton pregnant women with GDM diagnosed according to the JSOG criteria at 24–32 weeks of gestation who had delivered in the period of January 1, 2015, to December 31, 2019. In 2012, we introduced the SII regimen as a treatment option for women with GDM. Because we trained physicians to standardize the regimen in women with GDM from 2012 to 2014, we excluded cases encountered in this period, as it was considered a transitional period for the introduction of the regimen. Women with overt diabetes in pregnancy were also excluded owing to the possibility of their having pregestational diabetes. We also excluded patients who were treated with insulin outside of the center, those with a non-Japanese ethnicity, and those with a steroid medication or insulin allergy.

The present study was approved by the NHO Nagasaki Medical Center. Patients provided their informed consent for the collection of their clinical data for the purpose of research.

All pregnant women at the study institution underwent universal screening using a 50 g glucose challenge test at 24 to 28 weeks' gestation; those with test values of ≥7.49 mmol/L (135 mg/dL) underwent a diagnostic 75 g oral glucose tolerance test (OGTT) after overnight fasting. According to the JSOG criteria [9], women with one or more abnormal values above the cutoff (5.1 (92), 10.0 (180), and 8.5 (153) mmol/L (mg/dL) for fasting, 1 h and 2 h after a glucose load, respectively) were diagnosed as having GDM. Glycated hemoglobin (HbA1c) levels were also measured at the time of the diagnostic OGTT.

Regarding the management of GDM, dieticians started diet therapy soon after the diagnosis. Patients also underwent self-monitoring of blood glucose (SMBG) four times daily (at fasting (before breakfast) and 2 h after each meal) using the same SMBG equipment (OneTouchVerioVue®; LifeScan Japan). We used a target glucose levels < 5.27 mmol/L (95 mg/dL) at fasting and <6.66 mmol/L (120 mg/dL) at 2 h after each meal [9, 10]. Because postprandial capillary glucose values are known to be 20%-25% higher than the venous glucose values [11], we accepted <130 mg/dL as the postprandial target on the SMBG profile in the clinical setting. Fasting and postprandial blood glucose levels were assessed separately.

Insulin therapy was indicated if the SMBG value did not reach approximately ≥80% of the fasting and postprandial target glucose values, respectively [7]. Patients received dietary education at weekly to monthly intervals according to the characteristics of the subjects. We measured HbA1c and random glucose values at least once a month. Women being treated with insulin therapy were encouraged to continue SMBG until labor onset. In women with diet therapy alone, the attending physician decided whether the measurements should be abandoned or continued until labor onset. Attending physicians checked the SMBG memories in women whose SMBG report was discrepant with other clinical features, including the glucose values measured at every perinatal visit and sonographic fetal growth and amniotic fluid assessments.

At the beginning of insulin therapy, most patients received the SII regimen, which consisted of one to two daily injections of NPH insulin (Novolin N®). We used one daily injection of NPH insulin (10 to 20 units) before breakfast and added another at bedtime, if necessary. If the target values were still not reached, we switched to the MDI regimen with the combination of NPH insulin (at fasting and/or bedtime) and prandial injections of insulin aspart (NovoRapid®) at each meal. In the insulin regimens, insulin doses were adjusted according to the SMBG data in each case throughout pregnancy.

We interviewed baseline characteristics at the first perinatal visit during first trimester before the diagnosis or at the reference visit to the institute after the diagnosis. Prepregnancy obesity was defined as a BMI ≥ 25 kg/m^2^. Gestational weight gain (GWG) throughout pregnancy and before and after the diagnosis of GDM was examined. A family history (FH) of diabetes was defined as unspecified diabetes among second-degree relatives.

As indices of metabolic control, we used the SMBG measurements and HbA1c value at the diagnosis and the latest measurement before delivery (within four weeks before delivery). Regarding the SMBG values, we used the mean values of 7-day measurements at fasting (before breakfast) and those of 7-day measurements of three daily postprandial values, during the introduction period of the SMBG and 7 days before the last perinatal visit. As indices to assess the achievement of the target glucose goal, we used the mean values of fasting blood glucose < 95 mg/dL and the mean values of postprandial blood glucose < 130 mg/dL. Because, regarding the postprandial glucose, a value < 120 mg/dL is generally used as a target value worldwide, the achieving rate of the value was also compared between the groups. Hypoglycemic events were derived from medical records.

As perinatal outcomes, we compared the primary cesarean section rate, the incidence of hypertensive disorders of pregnancy (HDP), birthweight (BW), and BW *z*-score. Regarding the BW assessment, we used large-for-gestational age (LGA) and small-for-gestational age (SGA) infants, defined as those with a BW > 90th and <10 percentile, respectively, according to parity- and gender-specific Japanese BW curves [12]. Regarding neonatal complications, we defined clinical hypoglycemia and hyperbilirubinemia as that requiring glucose infusion and phototherapy, respectively.

We used Tukey's HSD test and Chi-square test to compare numerical and categorical variables, respectively, between the groups and *P* values < 0.05 were considered to indicate statistical significance.

## 3. Results

**Figure 1:**
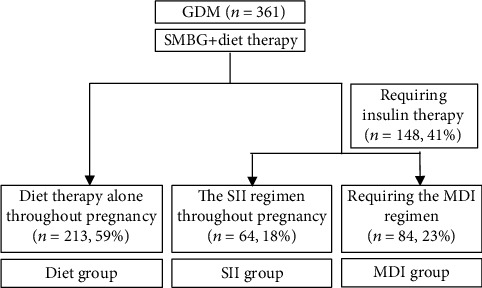
Flow diagram of patient inclusion in each group. GDM: gestational diabetes; SMBG: self-monitoring of blood glucose; SII: simple insulin injection; MDI, multiple daily insulin injection.

We included 361 patients; the mean maternal age and prepregnancy BMI were 33.8 ± 4.9 years and 23.2 ± 4.5 kg/m^2^, respectively. 41%, 28%, and 48% were nulliparous, prepregnancy obese (≥25 kg/m^2^), and with family history of diabetes, respectively. The mean gestational age at diagnosis of GDM were 26.0 ± 1.9 weeks' gestation. Among them, 213 (59%), 64 (18%), and 84 (23%) were treated with diet therapy alone (diet group), the SII regimen alone (SII group), and required the MDI regimen (MDI group), respectively (Figure 1). Forty-three percent of women requiring insulin therapy were treated with the SII regimen alone throughout pregnancy.

The maternal baseline characteristics and diagnostic OGTT results are summarized in Table 1. While the insulin group showed a statistically significant association with obesity, there were no significant differences in the baseline characteristics between the insulin groups. Regarding the diagnostic OGTT results, the fasting and 1 h and 2 h PG values did not differ between the insulin groups; however, the HbA1c levels were higher in the MDI group (Table 1).

Women in the SII group required less than half of a daily dose of total insulin and 24% less NPH insulin than the MDI group (Table 2). Neither the GWG throughout pregnancy nor that before or after the diagnostic OGTT differed between the groups. The HbA1c levels at delivery were very similar in each group. There were no hypoglycemic episodes requiring additional treatment during pregnancy in any of the insulin groups.

**Figure 2:**
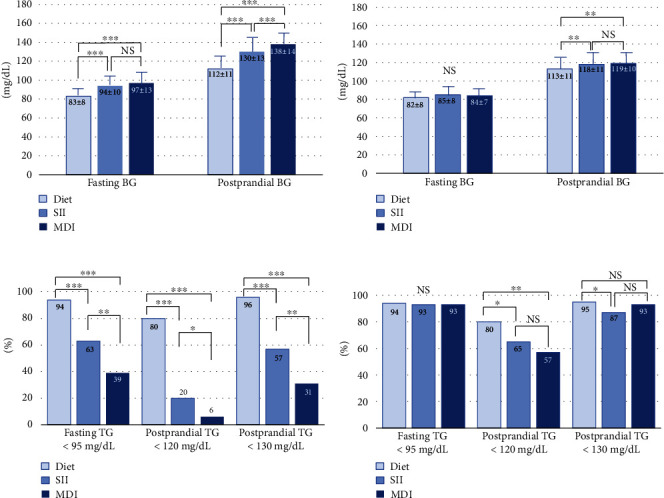
A comparison of the mean blood glucose values and the achieving rate of target glucose goals on SMBG at baseline and before delivery. ^∗^*p* < 0.05, ^∗∗^*p* < 0.005, and ^∗∗∗^*p* < 0.001. SMBG: self-monitoring of blood glucose; BG: blood glucose; SII: simple insulin injection; MDI: multiple dairy injection; TG: target glucose; NS: not significant.

The SMBG data are summarized in Table 3. The data at the time of the diagnosis were missed in 15, 4, and 4 cases in the diet, SII, and MDI groups, respectively. Regarding the SMBG data before delivery, one-third of women in the diet group were allowed to quit the SMBG measurements because of their good control, so we obtained the data measured in the 4 weeks before delivery in 142 cases in the group. The data before delivery were missed in four and three cases in the SII and MDI groups, respectively. Both fasting and postprandial values and the achievement rates of target glucose values at the time of the diagnosis differed significantly between the groups, demonstrating that the degree of glycemia in the SMBG was lowest in the diet group and highest in the MDI group (Table 3 and Figures 2(a) and 2(c)). As the baseline data, in the diet groups, 94% of women achieved the target glucose values at fasting and 80% and 96% achieved <120 mg/dL and <130 mg/dL postprandial, respectively. On the other hand, only 63%, 20%, and 57% in the SII group and 39%, 6%, and 31% in the MDI group achieved the target values, respectively.

Regarding the achievement of the target glucose goal on SMBG before delivery, the mean and achievement rate of fasting glucose values did not differ between the groups (Table 3 and Figures 2(b) and 2(d)). Regarding the postprandial glucose values before delivery, although the mean values in the diet group were still significantly lower than the insulin groups, the differences were small and did not differ within the insulin groups. Regarding the achievement rate of <120 mg/dL postprandial, the rates in the insulin groups were still significantly lower than that in the diet group. On the other hand, the rates did not differ within the insulin groups. The achievement rates of the postprandial values of <130 mg/dL effectively rose in both insulin groups. Although the rate in the SII group was significantly lower than the diet group, the rates did not differ within the insulin groups (Table 3 and Figures 2(b) and 2(d)).

The perinatal outcomes are summarized in Table 4. Although women in the insulin groups showed a significantly higher rate of HDP than those in the diet group, no marked difference was noted between the insulin groups. There were no significant differences in the perinatal outcomes other than the development of HDP between the groups.

## 4. Discussion

In this study, we found that, among women with GDM requiring insulin therapy, 43% were successfully treated with the SII regimen throughout pregnancy, with the rate of achieving target glucose values comparable to that in the MDI regimen. Women in the SII group also demonstrated similar perinatal outcomes to those in both diet and MDI groups, without excessive maternal weight gain. These findings support the efficacy of the SII regimen for some women with GDM in whom diet therapy alone failed to achieve target glucose values. Women who could be successfully treated with the SII regimen were characterized as being similarly obese to and having less severe glycemia than those in the MDI group.

Regarding insulin regimens using a single daily dose of NPH insulin alone for the treatment of GDM, some experts have reported the efficacy of a bedtime NPH regimen for women with fasting hyperglycemia, as expert opinions without clinical evidence [13–15]. There have been few studies addressing the efficacy of such NPH-only regimens for women with GDM with not only fasting but also postprandial hyperglycemia as a treatment option. Historically, O'Sullivan et al. [16, 17] reported that insulin therapy in women with GDM reduced macrosomic infants [16] and perinatal mortality [17] compared with diet therapy alone. Notably, in those reports, a fixed regimen with 10 units of NPH insulin alone was used. Later, Coustan et al. [18] proposed the concept of “prophylactic” insulin therapy to reduce macrosomia in GDM pregnancy. They used a uniform regimen involving the combination of a fixed dose of 20 units of NPH and 10 units of insulin before breakfast and found that the uniform regimen successfully reduced macrosomic infants compared with the diet-alone group. The prophylactic effect of the uniform combined fixed-dose insulin regimen was reproduced in a later study [19]. In those studies, however, the uniform regimen with a fixed insulin dose was used without SMBG measurements, so technically, the concept of the regimen was “prophylactic” and not “therapeutic.”

With the widespread use of the SMBG system during pregnancy in women with type 1 diabetes since the early 1980s, intensive tight glycemic control using the MDI regimen, which involves four daily injections instead of a single or double injection of insulin, has been widely used in the management of type 1 diabetic pregnancy [20]. Tight glycemic control using the MDI regimen with the SMBG system has been introduced for not only type 1 but also type 2 diabetes as well as GDM during pregnancy [21]. Nachum et al. [22] concluded that giving insulin four times rather than twice daily in pregnancy improved glycemic control and the perinatal outcomes in their randomized control trial (RCT). However, in the study, more than one-third of the patients were pregestational diabetes. More recently, two landmark RCTs [3, 4] demonstrated the efficacy of intervention in women with mild GDM using regimens consisting of either short-acting insulin alone or short-acting insulin and NPH in a basal/bolus regimen [23]. In these contexts, the MDI regimen appears to have become the standard insulin treatment in women with GDM, so a simple regimen involving NPH alone was abandoned.

However, while tighter glycemic control undoubtedly protects the health of both mother and infant, few studies have concluded that the MDI regimen is the best practice for all women with GDM who require insulin therapy. The MDI regimen consists of multiple injections of two different types of insulin—namely, intermediate/long-acting and rapid/ultrarapid insulin—and is thus more complicated than the SII regimen. In addition, such a complicated regimen can cause maternal anxiety during pregnancy [5]. Our finding that more than 40% of women requiring insulin therapy were successfully treated with the SII regimen alone may help reduce this anxiety.

Weight gain induced by intensive insulin therapy has long been recognized as a major problem in diabetes therapy [24, 25], especially with NPH insulin [26]. Weight gain causes adverse effects, including the manifestation of insulin resistance, hyperlipidemia, and blood pressure elevation, not only for nonpregnant adults but also for pregnant women. It occurs in the early stages of insulin introduction [25], and common reasons for the weight gain include caloric retention from a reduced urinary excretion of glucose and a reduction in the metabolic rate due to decreased hepatic glucose output [24, 25]. No report has yet described whether insulin therapy, especially regimens using NPH insulin alone, is associated with excessive GWG, although some investigators have reported that maternal GWG in women treated with insulin was greater than that in the metformin group [27, 28]. Because weight gain during pregnancy is a physiological phenomenon, it is difficult to distinguish insulin-mediated excessive weight gain from physiological weight gain during pregnancy. In our current study, we demonstrated that women treated with NPH alone were not associated with excessive GWG, with this population actually showing the least GWG among the three groups (Table 2). To our knowledge, this is the first report to compare the GWG between women treated with NPH insulin alone and those who received MDI insulin therapy among women with GDM. In addition, a recent study reported the mean GWG in uncomplicated pregnancies in Japanese population was 10.1 ± 3.7 kg [29]. We believe that the results of GWG (Table 2) were adequate, as the diet regimen generally includes 30% of caloric restriction for women with GDM.

The strength of this study is that we used SMBG data to evaluate glucose control. The SMBG measurements are used not only to decide the indication of insulin therapy but also to evaluate the tight glucose control required in women with GDM. Thus, in the clinical setting, SMBG is an essential and integral tool in the care of GDM. While the PG (but not the HbA1c) values at the diagnostic test differed slightly regarding the basal glycemia between the groups (Table 1), the SMBG indices at baseline clearly demonstrated that women with the SII regimen were more significantly hyperglycemic and had lower rates of achieving control than women in the diet group (Table 3), suggesting that the insulin therapy was rationally indicated. The baseline SMBG data also clearly indicated that the levels of hyperglycemia were significantly higher in the MDI group than in the SII group. Consequently, it is obvious that the level of baseline hyperglycemia in the SII group fell between that in the diet group and the MDI group. In addition to the perinatal outcomes, the SMBG indices clearly showed that the achievement rates of target glucose levels before delivery in the SII group were comparable to those in the MDI group (Table 3). Second, because the cases experienced during the first two years after the introduction of the SII regimen were excluded, we believe that, as a standardized protocol, this effectively minimized physician bias.

However, several limitations associated with the present study also warrant mention. First, the decision to switch the treatment from diet therapy alone to insulin therapy and from the SII to MDI regimen was left to the attending physicians, although the physicians shared the standard of achieving the target glucose value, with this achievement defined as reaching approximately 80% of the fasting and nonfasting target glucose values. Again, the baseline SMBG data plainly demonstrated that difference in the glycemic severity between the groups. In addition, the rate of insulin therapy in our study was 41% for women with GDM. The rate was relevant in the high-risk population managed in a tertiary perinatal care center [7, 8, 30–32]. Therefore, even though physicians' biases may have affected the results, such an influence was likely minimal. Regarding the postprandial target glucose, the achievement rate of <120 mg/dL in the insulin groups did not reach that in the diet group. Although a value of <120 mg/dL is generally recommended worldwide [11], the targets including both fasting and postprandial values were derived from empirical data of uncomplicated pregnant women, not evidence-based in the association of adverse perinatal outcomes [33]. In fact, the perinatal outcomes did not differ between the diet and insulin groups in our study. In addition, the targets were derived from venous sampling data, rather from SMBG data [33]. As described in Materials and Methods, it is a characteristic that postprandial values in the SMBG measurement are higher than those in the venous sampling, while those at fasting are similar to the venous values [11]. Therefore, we believe a value of <130 mg/dL is an acceptable target in the clinical setting. Finally, on top of having a small sample size, this study was conducted at a single tertiary center including women with only a Japanese ethnic background, so our results have limited adaptability to a general population. GDM is strongly associated with the development of type 2 diabetes after delivery, and East Asian populations, including the Japanese, have one of the highest incidences of type 2 diabetes and are among the least obese in the world [34, 35]. Therefore, our findings may contribute to some extent to the management of GDM, especially in East Asian populations.

In conclusion, we demonstrated that, among women with GDM who required insulin therapy because they failed to achieve the target glucose values with nutritional therapy alone, more than 40% were successfully treated with the SII regimen instead of the MDI regimen without any increase in adverse perinatal outcomes or excessive GWG. This simple procedure may ensure patient compliance with treatment, contribute to their safety, and reduce anxiety compared with the MDI regimen.

## Figures and Tables

**Table 1:** A comparison of the baseline characteristics and OGTT results between the groups.

	Diet alone (*n* = 213)	SII group (*n* = 64)	MDI group (*n* = 84)	*p* value (diet vs. SII)	*p* value (diet vs. MDI)	*p* value (SII vs. MDI)
Maternal age (years)	33.5 ± 4.9	33.1 ± 5.2	34.9 ± 4.7	0.79	0.09	0.07
Nulliparous (%)	92 (43)	21 (33)	34 (40)	0.14	0.67	0.34
Family history of diabetes (%)	98 (46)	33 (52)	42 (50)	0.13	0.53	0.44
History of GDM (%) per parous women	39/121 (32)	13/43 (30)	19/50 (38)	0.80	0.47	0.43
Prepregnancy BMI (kg/m^2^)	22.0 ± 3.7	25.1 ± 4.9	24.9 ± 4.9	<0.0001	<0.0001	0.97
Prepregnancy obesity (BMI ≥ 25 kg/m^2^)	40 (19)	27(42)	n(40)	0.0001	<0.0001	0.83
Results of the diagnostic OGTT						
Gestational age at OGTT (weeks)	26.0 ± 1.9	26.0 ± 2.0	25.8 ± 1.7	1.00	0.68	0.82
Fasting PG (mg/dL)	78 ± 9	85 ± 9	88 ± 10	<0.0001	<0.0001	0.21
1-h PG (mg/dL)	175 ± 24	179 ± 26	185 ± 22	0.55	0.0033	0.23
2-h PG (mg/dL)	155 ± 22	153 ± 26	163 ± 28	0.84	0.060	0.065
HbA1c (%)	5.2 ± 0.3	5.3 ± 0.3	5.5 ± 0.3	0.39	<0.0001	0.0021

Data were shown as mean ± SD or number (%). SII: simple insulin injection; MDI: multiple daily insulin injection; GDM: gestational diabetes; OGTT: oral glucose tolerance test; PG: plasma glucose.

**Table 2:** A comparison of the insulin therapy, GWG, and HbA1c at delivery between the groups.

	Diet alone (*n* = 213)	SII group (*n* = 64)	MDI group (*n* = 84)	*p* value (diet vs. SII)	*p* value (diet vs. MDI)	*p* value (SII vs. MDI)
Required insulin dose during pregnancy (U/day)	—	19.4 ± 7.7	46.0 ± 22.9	—	—	<0.0001
NPH insulin (U/day)	—	19.4 ± 7.7	25.6 ± 10.6	—	—	0.0084
Insulin aspart (U/day)	—	—	20.4 ± 16.7	—	—	—
GWG (kg)	7.7 ± 4.2	6.6 ± 4.1	7.7 ± 5.3	0.25	1.00	0.32
Pre-OGTT GWG (kg)	4.5 ± 3.3	3.6 ± 3.5	4.2 ± 3.9	0.17	0.70	0.63
OGTT-delivery GWG (kg)	3.1 ± 3.0	3.0 ± 3.3	3.6 ± 4.6	0.99	0.58	0.62
HbA1c before delivery (%)	5.5 ± 0.3	5.5 ± 0.4	5.5 ± 0.4	1.00	1.00	1.00

Data were shown as mean ± SD or number (%). GWG: gestational weight gain; SII: simple insulin injection; MDI: multiple daily insulin injection; GDM: gestational diabetes; BMI: body mass index; OGTT: oral glucose tolerance test.

**Table 3:** A comparison of the SMBG at baseline and before delivery between the groups.

	Diet alone (*n* = 213)	SII group (*n* = 64)	MDI group (*n* = 84)	*p* value (diet vs. SII)	*p* value (diet vs. MDI)	*p* value (SII vs. MDI)
Baseline SMBG data						
Number of cases	198	60	80			
Mean fasting BG (mg/dL)	83 ± 8	94 ± 10	97 ± 13	<0.0001	<0.0001	0.055
Mean postprandial BG (mg/dL)	112 ± 11	130 ± 13	138 ± 14	<0.0001	<0.0001	<0.0001
Achieved TG of fasting < 95 mg/dL	186 (94)	38 (63)	31 (39)	<0.0001	<0.0001	0.0040
Achieved TG of postprandial < 120 mg/dL	158 (80)	12 (20)	5 (6)	<0.0001	<0.0001	0.014
Achieved TG of postprandial < 130 mg/dL	190 (96)	34 (57)	25 (31)	<0.0001	<0.0001	0.0026
SMBG before delivery						
Number of cases	142	60	81			
Mean fasting BG (mg/dL)	82 ± 8	85 ± 8	84 ± 7	0.11	0.13	0.97
Mean postprandial BG (mg/dL)	113 ± 11	118 ± 11	119 ± 10	0.0027	0.0001	0.91
Achieved TG of fasting < 95 mg/dL	133 (94)	56 (93)	75 (93)	0.93	0.76	0.87
Achieved TG of postprandial < 120 mg/dL	114 (80)	39 (65)	46 (57)	0.026	0.0003	0.32
Achieved TG of postprandial < 130 mg/dL	136 (95)	52 (87)	75 (93)	0.020	0.31	0.24

Data were shown as mean ± SD or number (%). SMBG: self-monitoring of blood glucose; SII: simple insulin injection; MDI: multiple daily insulin injection; BG: blood glucose; TG: target glucose.

**Table 4:** A comparison of the perinatal outcomes between the groups.

	Diet alone (*n* = 213)	SII group (*n* = 64)	MDI group (*n* = 84)	*p* value (diet vs. SII)	*p* value (diet vs. MDI)	*p* value (SII vs. MDI)
Gestational age at delivery (weeks)	38.6 ± 1.8	38.1 ± 2.5	38.7 ± 0.9	0.11	0.94	0.11
Cesarean section (%)	49 (23)	16 (25)	22 (26)	0.74	0.56	0.87
Primary cesarean section (%)	29 (14)	5 (8)	13 (15)	0.21	0.68	0.16
Hypertension disorders in pregnancy (%)	3 (1)	4 (6)	6 (7)	0.031	0.009	0.83
Birthweight (g)	2945 ± 463	2925 ± 483	2997 ± 375	0.94	0.65	0.60
Male infants (%)	108 (51)	33 (52)	39 (46)	0.90	0.51	0.54
Birthweight *z*-score	−0.02 ± 1.03	0.06 ± 0.93	0.03 ± 1.01	0.82	0.93	0.97
LGA infants (%)	24 (11)	7 (11)	12 (14)	0.94	0.47	0.55
SGA infants (%)	24 (11)	4 (6)	6 (7)	0.24	0.29	0.83
Apgar score (1 min)	8.3 ± 1.0	8.3 ± 0.7	8.3 ± 0.7	0.96	0.99	0.93
Apgar score (1 min) ≤7 (%)	11 (5)	2 (3)	3 (4)	0.47	0.53	0.87
Apgar score (5 min)	9.0 ± 0.4	8.9 ± 0.5	9.0 ± 0.4	0.24	0.99	0.29
Apgar score (5 min) ≤7 (%)	0 (0)	1 (2)	0 (0)	0.07	—	0.25
Shoulder dystocia (%)	1 (0.6)	1 (1.7)	0 (0)	0.40	0.53	0.26
NICU admission (%)	33 (11)	13 (22)	12 (14)	0.40	0.59	0.25
Hypoglycemia	3 (2)	2 (3)	3 (4)	0.43	0.27	0.85
Hyperbilirubinemia (%)	21 (11)	7 (15)	12 (15)	0.99	0.38	0.49

Data were shown as mean ± SD or number (%). SII: simple insulin injection; MDI: multiple daily insulin injection; LGA: large-for-gestational age; SGA: small-for-gestational age; NICU: neonatal intensive care unit.

## Data Availability

The original data used to support the findings of this study are available via private communication with the corresponding author upon request.
